# Network Pharmacology Study on *Morus alba* L. Leaves: Pivotal Functions of Bioactives on RAS Signaling Pathway and Its Associated Target Proteins against Gout

**DOI:** 10.3390/ijms22179372

**Published:** 2021-08-29

**Authors:** Ki Kwang Oh, Md. Adnan, Dong Ha Cho

**Affiliations:** Department of Bio-Health Convergence, College of Biomedical Science, Kangwon National University, Chuncheon 24341, Korea; nivirna07@kangwon.ac.kr (K.K.O.); mdadnan1991.pharma@gmail.com (M.A.)

**Keywords:** *Morus alba* L. leaves, network pharmacology, gout, AKT1, γ-tocopherol, RAS signaling pathway

## Abstract

*M. alba* L. is a valuable nutraceutical plant rich in potential bioactive compounds with promising anti-gouty arthritis. Here, we have explored bioactives, signaling pathways, and key proteins underlying the anti-gout activity of *M. alba* L. leaves for the first-time utilizing network pharmacology. Bioactives in *M. alba* L. leaves were detected through GC-MS (Gas Chromatography-Mass Spectrum) analysis and filtered by Lipinski’s rule. Target proteins connected to the filtered compounds and gout were selected from public databases. The overlapping target proteins between bioactives-interacted target proteins and gout-targeted proteins were identified using a Venn diagram. Bioactives-Proteins interactive networking for gout was analyzed to identify potential ligand-target and visualized the rich factor on the R package via the Kyoto Encyclopedia of Genes and Genomes (KEGG) pathway on STRING. Finally, a molecular docking test (MDT) between bioactives and target proteins was analyzed via AutoDock Vina. Gene Set Enrichment Analysis (GSEA) demonstrated that mechanisms of *M. alba* L. leaves against gout were connected to 17 signaling pathways on 26 compounds. AKT1 (AKT Serine/Threonine Kinase 1), γ-Tocopherol, and RAS signaling pathway were selected as a hub target, a key bioactive, and a hub signaling pathway, respectively. Furthermore, three main compounds (γ-Tocopherol, 4-Dehydroxy-N-(4,5-methylenedioxy-2-nitrobenzylidene) tyramine, and Lanosterol acetate) and three key target proteins—AKT1, PRKCA, and PLA2G2A associated with the RAS signaling pathway were noted for their highest affinity on MDT. The identified three key bioactives in *M. alba* L. leaves might contribute to recovering gouty condition by inactivating the RAS signaling pathway.

## 1. Introduction

Gout is a common and complex arthritis disease, often causing severe pain, swelling, redness and tenderness due to joint inflammation [1]. Gout is characterized by a disorder of uric acid crystal accumulation in blood, and its deposition is a vital factor to induce acute inflammation within and around joints [2]. Commonly, gouty flare-ups are unexpected and intense, more frequent at night [3]. In general, males are more likely than females to undergo symptoms of gout. More males are diagnosed between 30 and 50 years old, and females are more prevalent after menopause [4]. A report expounds that gout prevention is fundamental through lifestyle changes such as limiting alcohol, relieving stress, regular exercise, and taking enough herbal and dairy products [5]. Another report shows that Traditional Chinese Medicine (TCM) is used to treat gout with satisfactory effect [6]. Even though researchers are conducting experiments, there are no complete drugs for patients with gout. Existing drugs such as colchicines, corticosteroids, and non-steroidal anti-inflammatory drugs (NSAIDs) are utilized as an amelioration strategy against gout [7]. These medications might show good efficacy for a short time; however, for longer time periods, gastrointestinal, nausea, vomiting, and even renal toxicity could occur [8]. Therefore, herbal medicine might be a favorable remedy to diminish negative side effects during administration.

*Morus alba* L. is commonly distributed in Japan, India, China, and Korea, frequently used to alleviate joint pain, kidney and liver complication, and type 2 diabetes mellitus by tradition. Due to its rich nutritional value, *M. alba* L. leaves are cultivated as food for silkworms which produce high-quality silk [9]. Apart from silk production, *M. alba* L. leaves are of great biological and pharmacological interest to researchers. They contain diverse polyphenolic compounds with potent antioxidant, anticancer, and anti-inflammatory effects [10,11,12]. Recent research revealed that methanolic extract of *M. alba* L. leaves notably diminished neutropenia, elevated phagocytic index, and evidently fostered immunomodulatory effects [13]. An animal experiment exposed that administration of *M. alba* L. leaves (70% methanolic extract) significantly reduced uric acid level in plasma and showed potent antioxidant activity in mice [14]. Another study concluded that *M. alba* L. leaves have potent anti-inflammatory and antioxidant activities that might be an excellent candidate to relieve gouty arthritis pain [15]. Moreover, *M. alba* L. leaves ethanolic extract is a potent inhibitor of xanthine oxidase (XO) enzyme associated directly with hyperuricemia [16]. Although many researchers proved to have promising analgesic, anti-inflammatory, and anti-arthritis potentials of *M. alba* L. leaves [17,18,19], however, the key bioactive compounds and mechanisms of *M. alba* L. leaves against gouty arthritis have not been established completely. *M. alba* L. leaves shed light on medicinal effects to alleviate symptoms of gout as well as a potent antagonist of XO. 

Hence, our goal is to substantiate bioactives and mechanisms of *Morus alba* (M. alba) L. leaves against gout as *Morus alba* (M. alba) L. leaves have been reported as an important herbal medicine to counteract gout. Our study used GC-MS analysis with ChemStation integrated algorithms to maximize the discovery of drug-likeness bioactives in *M. alba* L. leaves. 

**Figure 1:**
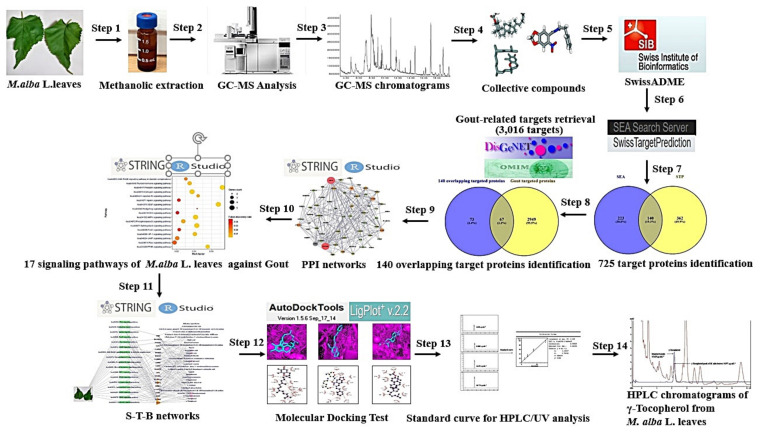
Workflow of network pharmacology analysis of *M. alba* L. leaves against Gout.

System biology has been focused on the multiple interactions in biology research from a whole viewpoint instead of adjusting to a single molecule [20]. For example, network pharmacology is utilized to identify multiple factors to interpret therapeutic compounds, toxicants, signaling pathways, hub proteins, and mechanisms of phytochemicals against various diseases [21,22]. With a systemic approach, network pharmacology can decode novel mechanism(s) of action which mainly focus on “multiple targets, multiple drugs” rather than “one target, one drug” [23,24]. The network pharmacology is a useful tool for constructing a compound-target-signaling pathway network through the overall perspective, and this holistic approach is very efficient for evaluating bioactive compounds [25,26]. However, in this study, network pharmacology was implemented to explore the bioactive constituents and mechanisms of *M. alba* L. leaves against gout. The brief analysis step of this study is displayed in Figure 1.

## 2. Results

### 2.1. Physicochemical Properties of Potential Chemical Compositions from M. alba L. Leaves

**Figure 2:**
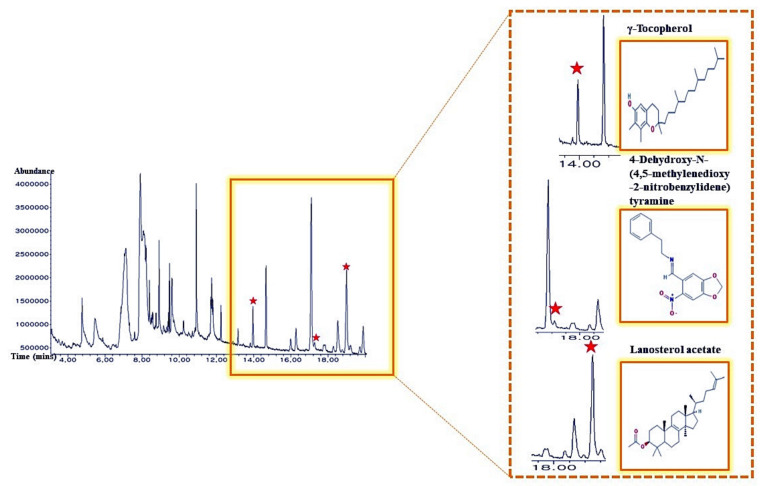
A typical GC-MS chromatogram of methanolic extract of *M. alba* L. leaves and indication of 3 main bioactives.

A total of 36 bioactives in *M. alba* L. leaves were identified via GC-MS analysis (Figure 2), and the name of compounds, retention time, peak area (%), Pubchem ID was presented in Table 1. All 36 bioactives were satisfied by Lipinski’s rule (Molecular Weight ≤ 500 g/mol; Moriguchi octanol-water partition coefficient ≤ 4.15; Number of Nitrogen or Oxygen ≤ 10; Number of NH or OH ≤ 5). The TPSA value of all bioactives was also accepted (Table 2). 

### 2.2. Overlapping Target Proteins between SEA and STP Associated with 36 Compounds

**Figure 3:**
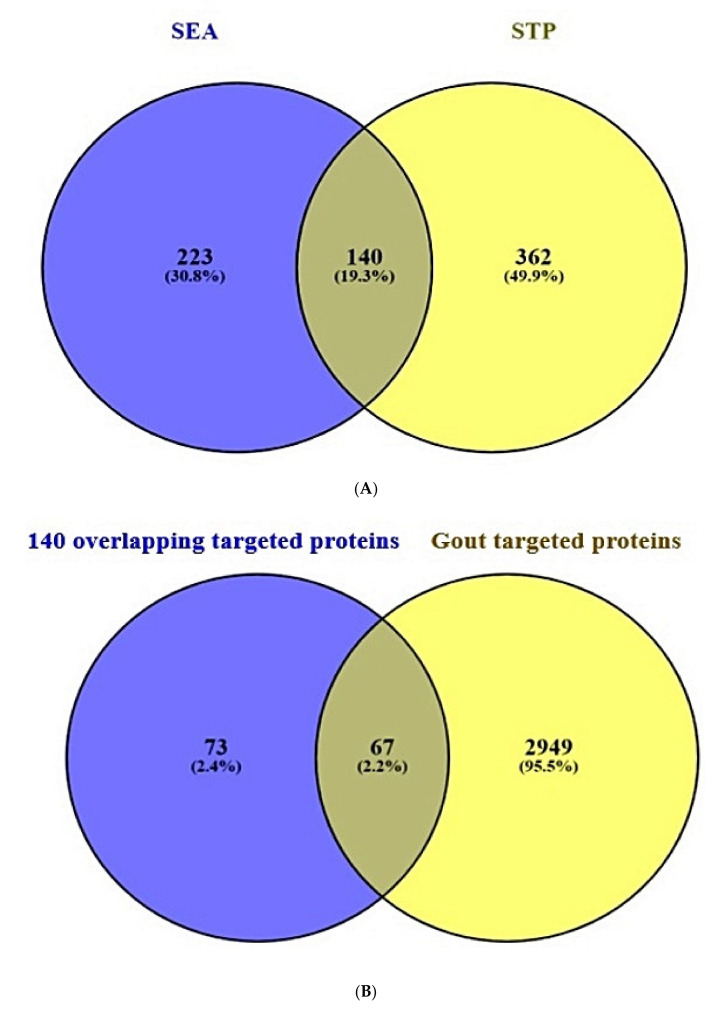
(**A**) Overlapping target proteins (140 target proteins) between SEA (223 target proteins) and STP (362 target proteins) (**B**) Overlapping target proteins between 140 overlapping target proteins from two databases (SEA and STP) and gout associated with target proteins (3016 target proteins).

A total of 363 target proteins from SEA and 502 target proteins from STP interacted with 36 compounds were extracted through SMILES format (Supplementary Table S1). Venn diagram showed that 140 target proteins were overlapping between the two public databases (Figure 3A).

### 2.3. Overlapping Target Proteins between Gout-Related Target Proteins and the 140 Overlapping Target Proteins

A total of 3016 target proteins connected to gout were selected by retrieving DisGeNET and OMIM databases (Supplementary Table S2). Venn diagram displayed that 67 overlapping target proteins were identified between the 3016 target proteins and the 140 overlapping target proteins (Figure 3B) and (Supplementary Table S2).

### 2.4. Protein-Protein Interaction from 60 Overlapping Target Proteins

**Figure 4:**
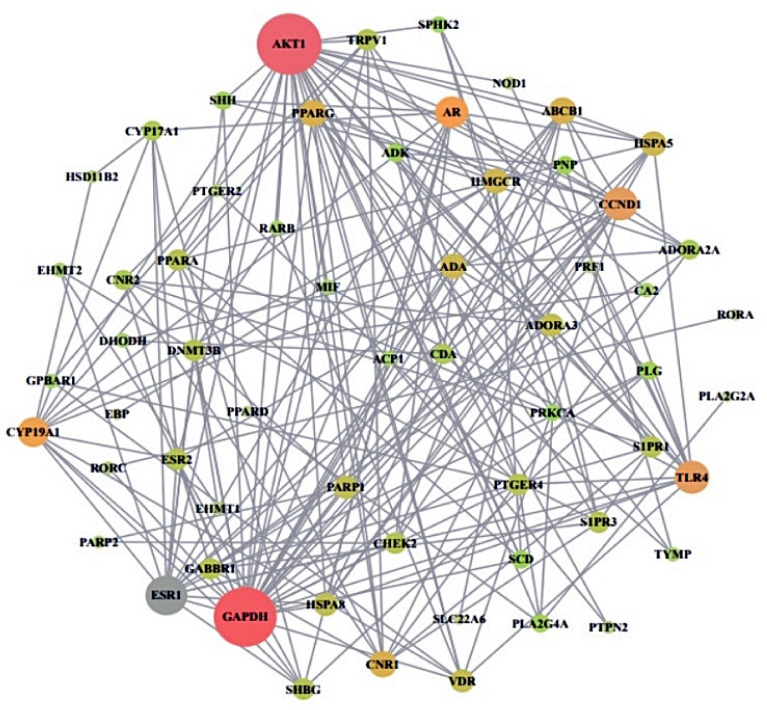
PPI networks of final overlapping 60 target proteins (60 nodes and 199 edges). Nodes: The number of networking proteins; Edges: Interactions between protein(s) and protein(s).

From STRING analysis, 60 out of 67 overlapping target proteins were closely interacted with each other, indicating 60 nodes and 199 edges (Figure 4). The removed 7 target proteins (HPSE, PAM, CA1, GSTK1, SLC5A2, GRK1, and BCHE) did not correlate within the overlapping 67 target proteins. In protein–protein interaction (PPI), the AKT1 target exhibited the highest degree (31) and is considered as a hub target protein (Table 3).

### 2.5. The 17 Signaling Pathways and Finding of a Hub Signaling of M. alba L. Leaves against Gout

**Figure 5:**
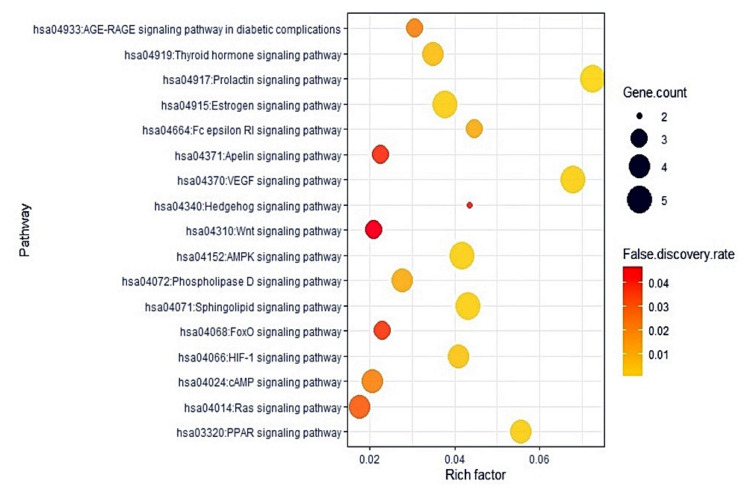
Bubble chart of 17 signaling pathways linked to the occurrence and progression of gout.

The KEGG pathway enrichment analysis demonstrated that 67 target proteins were associated with 17 signaling pathways (False Discovery Rate < 0.05). The 17 signaling pathways were directly related to gout development, exhibiting that these pathways might be the significant signal transduction of *M. alba* L. leaves against gout. The description of 17 signaling pathways was presented in Table 4. Additionally, a bubble plot suggested that the RAS (Renin Angiotensin System) signaling pathway might be a hub signaling pathway of *M. alba* L. leaves against gout (Figure 5).

### 2.6. A Signaling Pathway-Target Protein-Bioactive Networks

**Figure 6:**
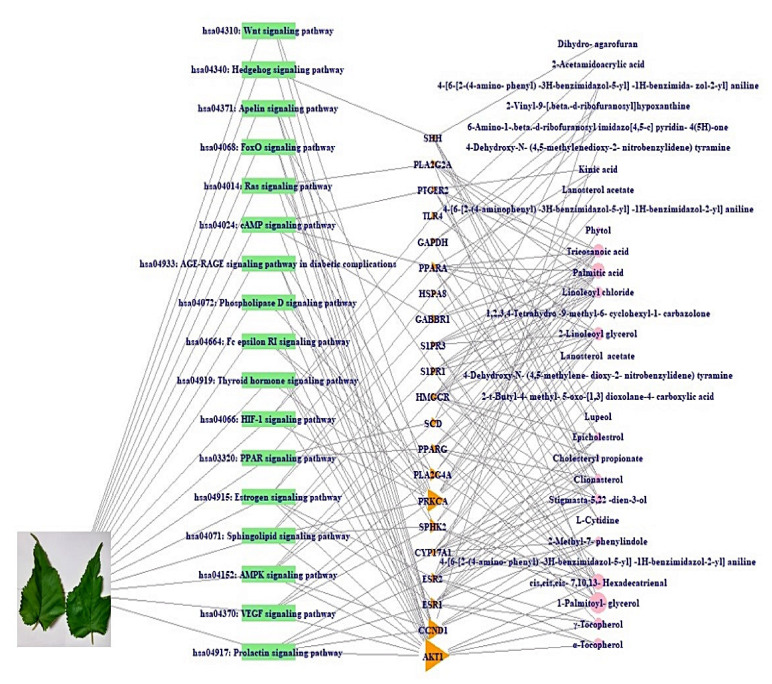
S-T-B networks of *M. alba* L. leaves.

A signaling pathway- target protein- bioactive (S-T-B) networks of *M. alba* L. leaves were displayed in Figure 6. There were 26 bioactives, 21 target proteins, and 17 pathways (64 nodes, 177 edges). The nodes represent a total number of bioactives, target proteins, and pathways. The edges indicate relationships of the three components. The S-T-B networks suggest that the network might interact with therapeutic efficacy against gout. The AKT1 is the most significant target with the highest degree value (14) among 17 signaling pathways related to 21 target proteins linked directly to the RAS signaling pathway.

### 2.7. MDT Results of 4 Target Proteins and 4 Compounds Related to RAS Signaling Pathway

**Figure 7:**
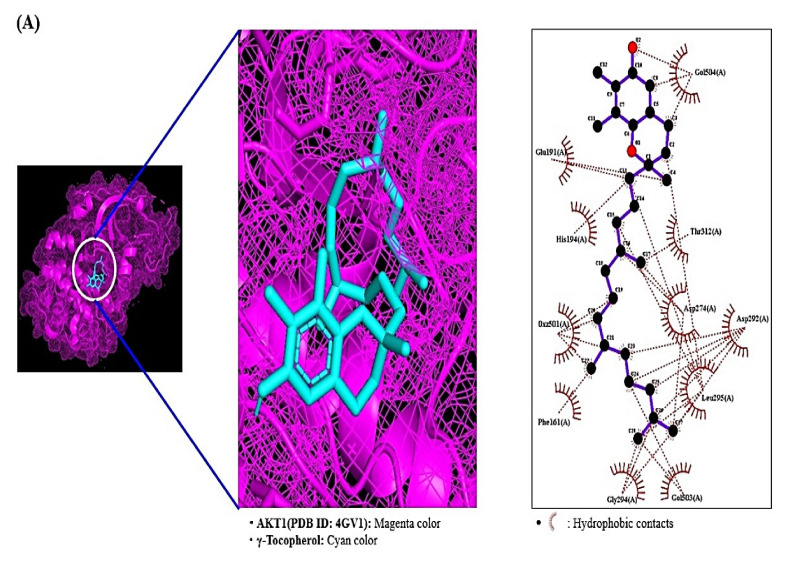

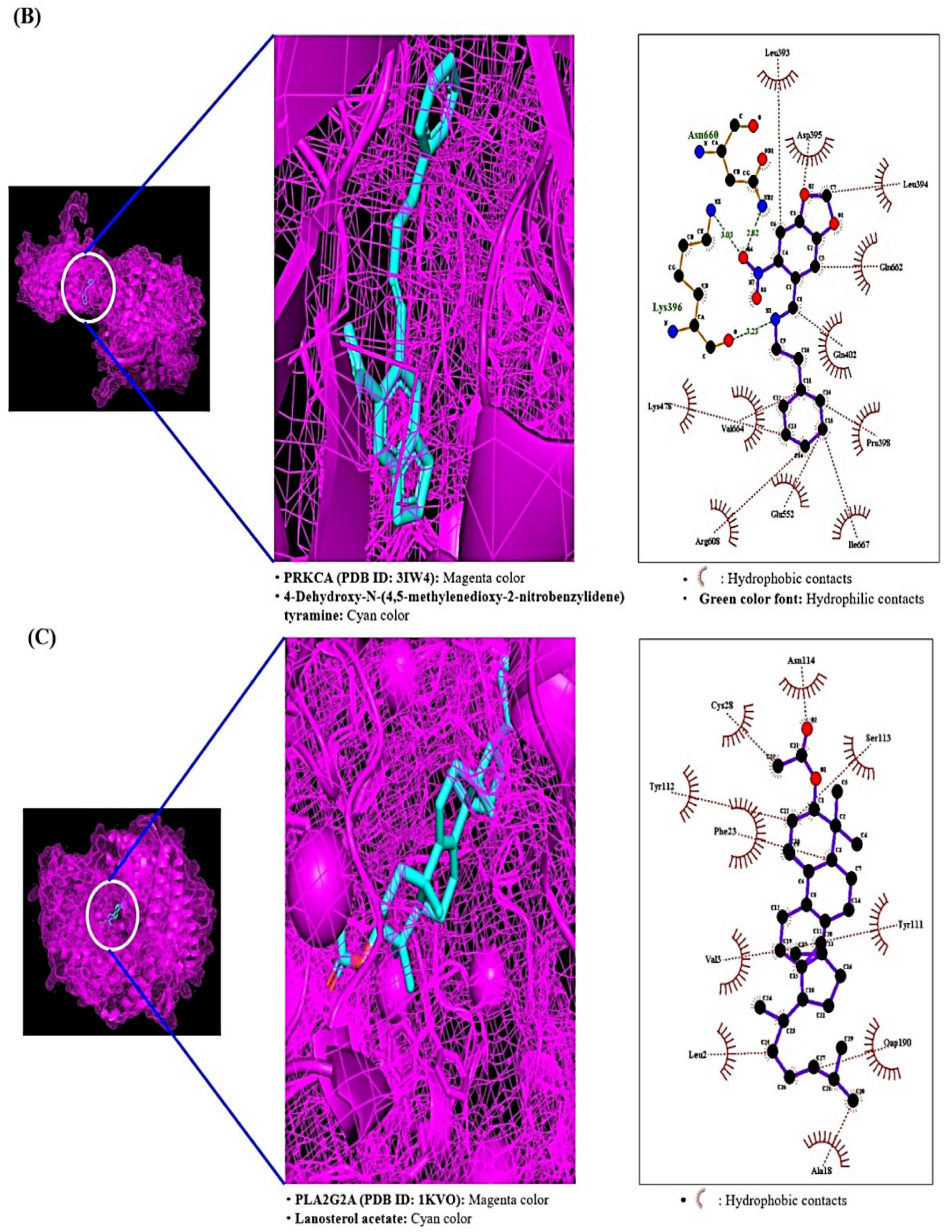
Molecular docking interaction between best docked compounds from SB and target proteins. (**A**) γ-Tocopherol on AKT1 (PDB ID: 4GV1). (**B**) 4-Dehydroxy-N-(4,5-methylenedioxy-2-nitrobenzylidene) tyramine on PRKCA (PDB ID: 3IW4). (**C**) Lanosterol acetate on PLA2G2A (PDB ID: 1KVO).

Through the analysis of SEA and STP database, it was revealed that AKT1 was linked to four compounds (γ-Tocopherol, α-Tocopherol, 1-Palmitoylglycerol, and cis, cis, cis-7, 10, 13-Hexadecatrienal), PRKCA was associated with seven compounds (1-Palmitoylglycerol, 2-Linoleoylglycerol, Linoleoyl chloride, Palmitic acid, Tricosanoic acid, Phytol, and 4-Dehydroxy-N-(4, 5-methylenedioxy-2-nitrobenzylidene) tyramine), PLA2G2A was linked to 5 compounds (1-Palmitoylglycerol, Linoleoyl chloride, Palmitic acid, Tricosanoic acid, and Lanosterol acetate), PLA2G4A was linked to 5 compounds (2-Linoleoylglycerol, Linoleoyl chloride, Palmitic acid, Tricosanoic acid, and cis, cis, cis-7, 10, 13-Hexadecatrienal. The MDT was performed to evaluate these four proteins’ binding energy against each related gene, individually. The docking figures were depicted in Figure 7A–C. The MDT score of four ligands on AKT1 protein (PDB ID: 4GV1) was analyzed in the “*Homo Sapiens*” mode. It was observed that γ-Tocopherol (−7.3 kcal/mol) docked on AKT1 exposed the most excellent binding energy, followed by α-Tocopherol (−7.0 kcal/mol), 1-Palmitoylglycerol (−6.9 kcal/mol), and cis-cis-cis-7,10,13-Hexadecatrienal (−4.8 kcal/mol). The detailed information was enlisted in Table 5. The MDT score of seven ligands on PRKCA protein (PDB ID: 3IW4) was conducted in the “*Homo Sapiens*” mode. It was exposed that 4-Dehydroxy-N-(4,5-methylenedioxy-2-nitrobenzylidene)tyramine (−8.4 kcal/mol) docked on PRKCA manifested the most significant binding energy, followed by 2-Linoleoylglycerol (−6.9 kcal/mol), 1-Palmitoylglycerol (−6.6 kcal/mol), Tricosanoic acid (−6.5 kcal/mol), Phytol (−5.6 kcal/mol), Palmitic acid (−5.0 kcal/mol), and Linoleoyl chloride (−4.8 kcal/mol). The detailed information was shown in Table 6. The MDT score of five ligands on PLA2G2A protein (PDB ID: 1KVO) was identified in the “*Homo Sapiens*” mode. It was exhibited that Lanosterol acetate (−8.4 kcal/mol) docked on PLA2G2A revealed the highest binding energy, followed by 1-Palmitoylglycerol (−6.8 kcal/mol), Tricosanoic acid (−5.9 kcal/mol), Palmitic acid (−5.4 kcal/mol), and Linoleoyl chloride (−4.8 kcal/mol). The docking results were enlisted in Table 7. The MDT score of five ligands on PLA2G4A protein (PDB ID: 1BCI) was evaluated in the “*Homo Sapiens*” mode. It was revealed that 2-Linoleoylglycerol (−4.9 kcal/mol) showed the greatest binding energy, followed by cis-cis-cis-7, 10, 13 Hexadecatrienal (−4.1 kcal/mol), Linoleoyl chloride (−4.0 kcal/mol), Tricosanoic acid (−3.6 kcal/mol), and Palmitic acid (−3.3 kcal/mol). Interestingly, the MDT score of 5 compounds (D1-D5) on PLA2G4A demonstrated invalid affinity scores (>−6.0 kcal*mol^−1^) [42]; accordingly, we did not regard them as potential bioactives against gout. The docking detail information was presented in Table 8.

### 2.8. Linearity of Standard γ-Tocopherol

**Figure 8:**
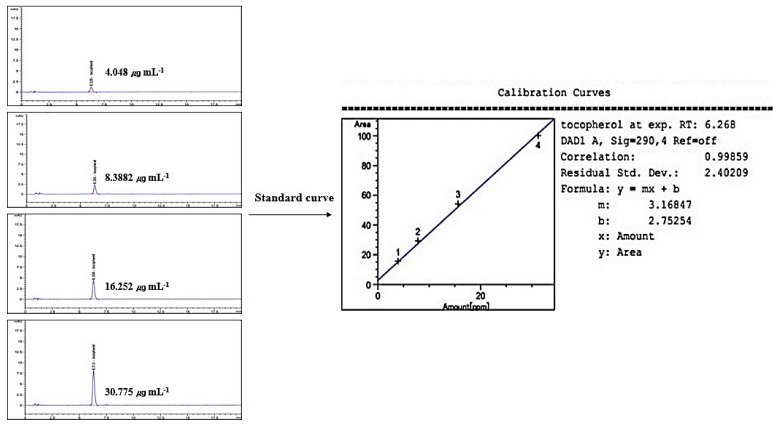
Standard curve for HPLC/UV analysis of γ-Tocopherol (wavelength: 290 nm).

Linearity was evaluated by the standard curve, determined by 4 different concentrations of γ-Tocopherol dissolved in MeOH. The peak area was obtained to calculate the correlation coefficient of square linear regression analysis. The linearity of peak area responses versus concentrations was identified in the range of 4.048 mg mL^−1^ to 30.775 mg mL^−1^ (*r* = 0.99859, *n* = 4) (Figure 8).

### 2.9. The Identification of γ-Tocopherol from M. alba L. Leaves

**Figure 9:**
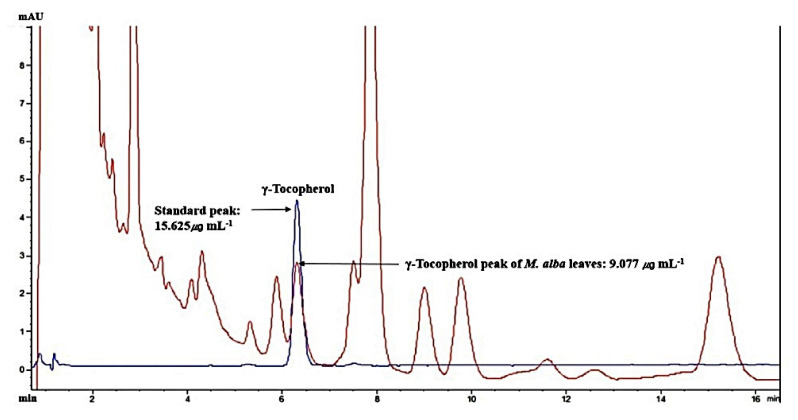
Overlapping HPLC chromatograms obtained by standard γ-Tocopherol (blue curve) and γ-Tocopherol (red curve) in *M. alba* L. leaves MeOH extraction, wavelength: 290 nm.

The retention time of *γ-Tocopherol* was 6.271 min in the HPLC analysis system, which overlapped exactly with the standard solution. The *γ-Tocopherol* amount was 9.077 mg mL^−1^ in *M. alba L. leaves* MeOH extraction (20 mg mL^−1^) (Figure 9). The ratio of *γ-Tocopherol* was comprised around 0.045% in HCLLs MeOH extract.

### 2.10. Toxicological Properties of Selected Key Compounds

Additionally, toxicological properties of the key three compounds (γ-Tocopherol, 4-Dehydroxy-N-(4,5-methylenedioxy-2-nitrobenzylidene) tyramine, and Lanosterol acetate) were predicted by admetSAR online tool. Our result indicated that the three compounds did not reveal Ames toxicity, carcinogenic properties, acute oral toxicity, and rat acute toxicity properties (Table 9).

## 3. Discussion

AKT1 is the highest degree (31) in PPI and the greatest degree (14) among 21 target proteins associated with 17 signaling pathways. Based on each target’s degree value, AKT1 was regarded as the hub target of *M. alba* L. leaves against gout. A report demonstrated that AKT1-knockout-mice exposed noticeably reduced edema comparable in control groups; the inhibition of inflammation was related to a significant reduction in neutrophil and monocyte [43]. Among 26 compounds in *M. alba* L. leaves, γ-Tocopherol with the strongest affinity on AKT1 was the uppermost bioactive against gout. Vitamin E reported in nature consists of four alpha (α), beta (β), gamma (γ), and delta (δ)- Tocopherol, both α-Tocopherol and γ-Tocopherol have anti-inflammatory efficacy in vitro and in vivo, substances with γ-Tocopherol have stronger potency than α-Tocopherol alone [44,45,46,47]. Among 17 signaling pathways, RAS signaling pathway was a hub signaling pathway based on rich factor with the lowest value on STRING analysis. The RAS signaling pathway can regulate IL-6 secretion; specifically, IL-6 production is associated with inflammation, immunity, and bone metabolism [48]. Network pharmacology analysis expounded that 17 signal pathways of *M. alba* L. leaves against gout were related to 26 compounds out of 36 compounds detected by GC-MS, including six prenol lipids (α-Tocopherol, γ-Tocopherol, Lupeol, Lanosterol acetate, Phytol, and Dihydroagarofuran). The ratio of prenol lipids to 26 compounds was close to 25%, suggesting that prenol lipids were more significant than any other kind of compound for the amelioration of *M. alba* L. leaves on gout. It was reported that prenol lipids are involved in cell proliferation and differentiation in smooth muscle cell [49]. Other studies suggested that prenol lipids are the important regulator for inflammation and bone health [50,51,52].

The PPI displayed that 17 signaling pathways were directly associated with gout occurrence and development, implying that the 17 signaling pathways might be the molecular mechanisms of *M. alba* L. leaves against gout. Thus, the 17 signaling pathways connected to gout were briefly discussed as follows. PPAR (Peroxisome Proliferator-Activated Receptor) signaling pathway: PPAR–γ (Peroxisome Proliferator-Activated Receptor-Gamma) expression on monocytes aggravated gouty arthritis and accelerated cytokine secretion [53]. RAS (Renin-Angiotensin System) signaling pathway: Uric acid is a leading causative element of gout, inducing oxidative stress via RAS activation [54]. It is evident that the inactivation of RAS may diminish the inflammatory level of gout. cAMP (cyclic Adenosine MonoPhosphate) signaling pathway: The increased cAMP level debilitated the MSU (Mono Sodium Urate)-induced activation of the Nod-like receptor protein 3 (NLRP3) signaling pathway, indicating the vital role of cAMP in the regulation of P2Y_14_ receptor (P2Y_14_R)-mediated gouty arthritis [55]. HIF-1 (Hypoxia Inducible Factor-1) signaling pathway: MSU crystals increased the gene expression level of Hypoxia Inducible Factor -1 α (HIF-1α) in Fibroblast-Like Synoviocytes (FLS), and its expression in FLS might be an indication of inflammation [56]. FoxO (Forkhead box O) signaling pathway: FoxO is a transcription factor to modulate AKT for IL-RA (Interleukin Receptor Antagonist) inhibition, which is an upstream controller to secrete cytokines [57]. Sphingolipid signaling pathway: Sphingolipids can ameliorate synovial inflammation and restore injured joints’ responses [58]. Phospholipase D signaling pathway: Microcrystals–induced arthritis triggers phospholipase D in human neutrophils, and its activation was partially intolerance to colchicine used as gout treatment [59]. AMPK (AMP-activated Protein Kinase) signaling pathway: The consistent AMPK activation could diminish lysosomal NKA (Na^+^-K^+^-ATPase) breakdown and sustain NKA function, thus relieving NKA inflammation and preserving tubular cells from high Uric acid-induced renal tubular damage [60]. Wnt (*Wingless*-*INT*) signaling pathway: Wnt signaling molecules and in vivo and in vitro animal studies suggest that Wnt signaling is an important therapeutic target for osteoarthritis, and the target tissues of Wnt signaling may be articular cartilage, synovium, and subchondral bone [61]. Hedgehog signaling pathway: The aberration of Hedgehog signaling regulation results in multiple bone diseases like heteroplasis, and thus, Hedgehog might be a promising biomarker for abnormal bone cartilage development [62]. VEGF (*Vascular Endothelial Growth Factor)* signaling pathway: A report suggested that VEGF counteracted properly pain responses and/or enhanced cartilage degeneration, synovitis, and osteophyte formation. Moreover, inhibition of VEGF signaling results in reduced pain [63]. Apelin (APLN) signaling pathway: APLN can control peripheral pain sensitivity sustained by APJ (APLN receptor) [64]. FcεRI (Fc epsilon RI) signaling pathway: IgE (Immunoglobulin-E) mediated by FcεRI signaling pathway inhibits bone remodelling due to mast cell activation, implicating gouty arthritis occurrence [65]. Estrogen signaling pathway: Estrogen treatment in rats has led to a dose-dependent cartilage weakness and a decrease in the extracellular matrix [66]. Prolactin signaling pathway: Prolactin treatment in rats diminished joint swelling, expanded trabecular bone area, reduced osteoclast density as well as protected bone loss in inflammatory arthritis [67]. Thyroid signaling pathway: Hyperthyroidism decreases the proinflammatory activities of monocytes and macrophages, which aggravate inflammation on gouty arthritis [68,69,70]. AGE-RAGE (Advanced Glycation End products- Receptor of Advanced Glycation End products) signaling pathway in diabetic complications: A study suggested that uric acid overexpressed the AGE-RAGE, which increased secretion of the inflammatory cytokine [71]. These signaling pathways imply interaction of multi-compound, multi-target, and multi-mechanism in the anti-gout activity of *M. alba* L. leaves.

**Figure 10:**
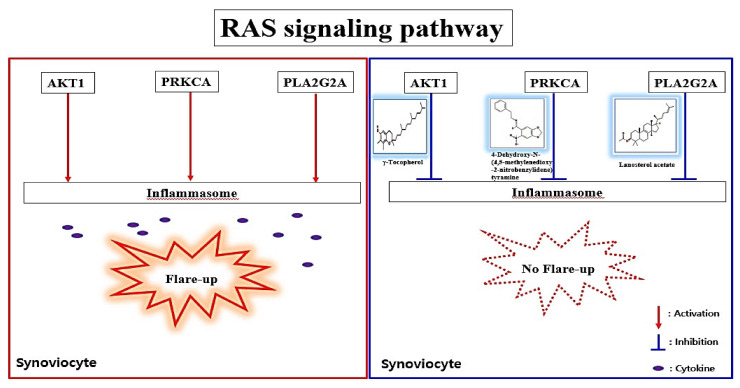
Summary figure of key findings in the study.

Based on MDT, a hub bioactive of *M. alba* L. leaves against gout is γ-Tocopherol which had the strongest affinity on AKT1 (considered as a hub target against gout). The AKT1 of *M. alba* L. leaves against gout was directly connected to 14 out of 17 signaling pathways by the RAS signaling pathway, suggesting that the RAS signaling pathway might be a hub signaling pathway *M. alba* L. leaves against gout. A bone joint is the central disease region in gout patients, and its inflammatory arthritis is characterized by swelling, tenderness, and redness [72]. Moreover, gout patients indicated low anti-apoptotic target proteins (Bcl-2, Bcl-X_L_) in synovial T cells, which is clear evidence of immunocompromised condition during gouty arthritis [73]. Recently, an animal experiment showed that colchicine (a common drug for gout) on macrophage in a mouse brain inhibits the RAS gene family with the inhibition of IL-1β (Interleukin 1 beta) [74]. The RAS inhibitors might promote anti-arthritis immunity in addition to targeting the macrophage cell’s dependency on the RAS signaling [75]. It is clear evidence that inflammatory reaction around bone cartilage might be to control via RAS signaling pathway. A report concluded that γ-Tocopherol is vital in inhibiting inflammation-associated diseases like rheumatoid arthritis, asthma, and even hepatitis [44]. It is evident that γ-Tocopherol is bound to AKT1 (a hub target on RAS signaling pathway) to foster anti-gout arthritis by blocking the RAS signaling pathway. The PRKCA is related to chronic pain of human osteoarthritis and over-expressed mRNA abundance levels in an osteoarthritis rat model [76,77]. However, it is not reported that 4-Dehydroxy-N-(4, 5-methylenedioxy-2-nitrobenzylidene) tyramine on PRKCA functioned as an anti-inflammatory effect in immunology. The PLA2G2A over-represented in synovial fluid samples of gouty arthritis patients was identified via liquid chromatography tandem mass spectrometry (LC–MS/MS), compared to Ankylosing Spondylitis (AS) [78]. We suggest that lanosterol acetate on PLA2G2A might be a potent antagonist by blocking the RAS signaling pathway. The PLA2G4A plays an essential role in regulating inflammatory response with Cyclooxygenase-2 (COX-2) activation mirrored eicosanoid biosynthesis [79]. However, compounds of *M. alba* L. leaves related to PLA2G4A did not show attractive docking scores (>−6.0 kcal/mol). The cut-off of AutoDock Vina program was considered as active molecules (binding affinity value < −6.0 kcal/mol) [42]. Furthermore, according to the highest MD, three bioactives have been selected, specifically γ-Tocopherol, 4-Dehydroxy-N-(4,5-methylenedioxy-2-nitrobenzylidene) tyramine, and Lanosterol acetate, to clarify their physicochemical and toxicological properties. If any bioactives are not accepted by Lipinski’s rule, it will not be evaluated as good oral bioavailability [80,81]. Our research showed that none of the bioactives, except “4-Dehydroxy-N-(4,5-methylenedioxy-2-nitrobenzylidene) tyramine”, violated Ames, which demonstrates good oral bioavailability. The study of toxicology suggested that none of the bioactives constitute a risk of Ames toxicity, carcinogenic properties, acute oral toxicity, and rat acute toxicity. To sum up, all three bioactives could be potential drug candidates with good oral bioavailability against gout. Therefore, the key mechanism of *M. alba* L. leaves against gout might be to suppress the inflammasomes in synovial fluids by inhibiting AKT1 by γ-Tocopherol, PRKCA by 4-Dehydroxy-N-(4,5-methylenedioxy-2-nitrobenzylidene) tyramine, and PLA2G2A by Lanosterol acetate on the RAS signaling pathway (Figure 10).

## 4. Materials and Methods

### 4.1. Plant Material Collection and Classification

The *M. alba* L. leaves were collected from (Latitude: 36. 666700, Longitude: 128. 510729, Gyeongsangbuk-do, Republic of Korea, in August 2020, the plant was identified by Dr. Dong Ha Cho, Plant biologist and Professor, Department of Bio-Health Convergence, College of Biomedical Science, Kangwon National University. A voucher number (CRT 103) has been stored at Kenaf Corporation in the Department of Bio-Health Convergence, and the material can be used only for research purposes.

### 4.2. Plant Preparation, Extraction

The experimental *M. alba* L. leaves were harvested in May 2020 before fructifying. The growth stage of their leaves was fully grown at 8~12 cm. The dried leaves (20 g) at room temperature (20~22 °C) for 7 days were soaked in 500 mL of methanol (Daejung,Siheung city, Korea). The extraction was carried out in a sealed bottle for 3 days and repeated 3 times at room temperature (20~22 °C). During extraction, the sample was shaken several times to increase the yield rate. The methanol was evaporated using a vacuum evaporator (IKA, Staufen city, Germany). The evaporated sample was dried under a hot water bath (IKA, Staufen city, Germany) at 40 °C.

### 4.3. GC-MS Condition

The analysis was carried out using the GC-MS system (Agilent 7890A, 5975C Agilent Technologies Inc., Santa Rosa, CA, USA) equipped with a DB-5 capillary column (30 m × 0.25 mm × 0.25 μm). Firstly, the GC-MS instrument was maintained at a temperature of 100 °C for 2.1 min. The temperature rose to 300 °C at the rate of 25 °C/min and was maintained for 10 min at the end of this period. Injection port temperature and helium flow rate were maintained as 250 °C and 1.5 mL/min. The samples injected in split mode at 10:1, and the ionization voltage was 70 eV. MS scan range was set at 35–550 (m/z), and the fragmentation patterns of mass spectra compared in W8N05ST Library MS database. The relative peak area of each compound in the chromatogram was calculated on each compound percentage. ChemStation integrated algorithms were used as the concept of integration (analyzed 11 February 2021) [82].

### 4.4. GC-MS Compounds in M. alba L. Leaves and Lipinski’s Rule

The species of chemical compounds from *M. alba* L. leaves were detected through GC-MS. The compounds identified by GC-MS input into the PubChem (https://pubchem.ncbi.nlm.nih.gov/) (accessed on 17 February 2021) to identify SMILES (Simplified Molecular Input Line Entry System). The identification of the “Drug-likeness” property is based on Lipinski’s rule in SwissADME (http://www.swissadme.ch/) (accessed on 20 February 2021) [83]. Moreover, the topological polar surface area (TPSA) value evaluates the ligands’ cell permeability identified by SwissADME; generally, its permeability is typically limited when the TPSA value exceeds 140 Å^2^ [84].

### 4.5. Target Proteins Associated with Bioactives or Gout

The bioactives accepted by Lipinski’s rule input SMILE format into the two databases: SEA (Similarity Ensemble Approach) (http://sea.bkslab.org/) (accessed on 22 February 2021) [85] and STP (SwissTargetPrediction) (http://www.swisstargetprediction.ch/) (accessed on 22 February 2021) [86] with “*Homo Sapiens*” setting. The target proteins—compounds interaction obtained by the two cheminformatics have been confirmed as powerful tools to be validated experimentally: SEA showed an accuracy rate of 80% out of novel drug candidates, and STP demonstrated that predictive target proteins of cudraflavone C was found via STP, thereby, validated by experiment [87,88]. Taken together, we assured that the novel new target(s) and mechanisms(s) against gout would be discovered by utilizing the validated data. Target proteins involved in gout were identified by two bioinformatics-DisGeNET (https://www.disgenet.org/search) (accessed on 2 March 2021) and OMIM (https://www.ncbi.nlm.nih.gov/omim) (accessed on 3 March 2021). The overlapping target proteins between drug-likeness compounds of *M. alba* L. leaves and gout-targeted proteins were identified and visualized on the Venn diagram by VENNY 2.1 (https://bioinfogp.cnb.csic.es/tools/venny/) (accessed on 4 March 2021).

### 4.6. Network Construction of Overlapping Target Proteins and Identification of Rich Factor

Final overlapping target proteins were visualized through STRING (https://string-db.org/) analysis (accessed on 5 March 2021) [89]. The overlapping target proteins were closely co-expressed, and thus, signaling pathways associated with the overlapping target proteins were conceptualized by R Package bubble chart analysis. Based on rich factor and false discovery rate (FDR < 0.05), a hub signaling pathway of *Morus alba* (*M. alba*) L. leaves against gout were selected.

### 4.7. A Signaling Pathway- Target Protein- Bioactive (S-T-B) Networks Construction

The S-T-B networks were used to construct a size map, based on degree of values. In the network map, green rectangles (nodes) stood for signaling pathways; pink triangles (nodes) stood for target proteins, and orange circles (nodes) stood for bioactives; its circle size represented degree value. The size of pink triangles represented the number of connectivity with signaling pathways; the size of orange circles represented the number of connectivity with target proteins. The merged networks were constructed by using RPackage.

### 4.8. Bioactives Preparation for MDT on a Hub Signaling Pathway

The bioactives connected to a hub signaling pathway were converted .sdf from PubChem into .pdb format using Pymol, finally, they were converted into .pdbqt format through Autodock.

### 4.9. Target Proteins Preparation for MDT

Four target proteins of gout i.e., AKT1 (PDB ID: 4GV1), PRKCA (PDB ID: 3IW4), PLA2G2A (PDB ID: 1KVO), PLA2G4A (PDB ID: 1BCI) were identified on STRING through RCSB PDB (https://www.rcsb.org/) (accessed on 7 March 2021). The proteins selected as .PDB format was converted into .pdbqt format via Autodock (http://autodock.scripps.edu/) (accessed on 7 March 2021).

### 4.10. MDT of Bioactives on Target Proteins Associated with a Hub Signaling Pathway

The ligand molecules were docked with target proteins utilizing autodock4 by setting-up 4 energy range and 8 exhaustiveness as default to obtain 10 different poses of ligand molecules [90]. The center of each target was AKT1 (x = 6.313, y = −7.926, z = 17.198), PRKCA (x = −14.059, y = 38.224, z = 32.319), PLA2G2A (x = −48.436, y = 71.878, z = 47.001), PLA2G4A (x = −0.058, y = 0.077, z = 0.285). The active site’s grid box size was x = 40 Å, y = 40 Å, z = 40 Å. The 2D binding interactions was identified through LigPlot+ v.2.2 (https://www.ebi.ac.uk/thornton-srv/software/LigPlus/) (accessed on 9 March 2021). After docking, ligands with the lowest Gibbs free energy were selected to visualize the ligand-protein docking in Pymol.

### 4.11. Chemicals and Reagents for HPLC Analysis

Standard γ-Tocopherol was purchased from Sigma Aldrich (St. Louis, MO, USA). HPLC grade MeOH was obtained from Burdick & Jackson. Ultrapure water obtained using a Milli-Q UF-Plus instrumentation (Millipore, MA, USA) was utilized to prepare all solutions for the method.

### 4.12. Instrumentation and Chromatographic Conditions

HPLC Agilent 1260 series chromatographic instrumentation was used for this research. Data was collected and processed with Agilent 1260 chemstation. The HPLC system was equipped with an injection valve, quaternary gradient pump system, and UV dual λ absorbance detector. Chromatographic separation was performed on a C18 column 4.6 × 150 mm, 3.5 *μ*m. The mobile phase was isocratic MeOH 98% (98:2, *v*/*v*, MeOH: water) at a flow rate of 2 mL min^−1^. Its analysis performed at ambient temperature, and detection was made at 290 nm. The injected volume was 20 *μ*L.

### 4.13. Preparation of Standard Solution

A stock solution of standard (γ-Tocopherol) was prepared in MeOH. The prepared stock solution concentration was made 3.906, 7.813, 15.626, and 31.250 ppm to plot the standard curve.

### 4.14. Preparation of Plant Extraction for HPLC Analysis

The 600 mg of *M. alba* L. leaves MeOH extraction was taken in a flask, 30 mL of MeOH was added and kept for 3 h. After shaking several times, the extraction was left for 5 days at room temperature. The solution of the flask was filtered through a Whatman No. 1 filter paper. The filtered solution was passed through a 0.2 *μ*m syringe filter and performed HPLC analysis.

### 4.15. Toxicological Properties Prediction by admetSAR

Toxicological properties of the key compounds were established using the admetSAR web-service tool (http://lmmd.ecust.edu.cn/admetsar1/predict/) (accessed on 12 March 2021) because toxicity is a central element to develop new drugs. In the current study, Ames toxicity, carcinogenic properties, acute oral toxicity, and rat acute toxicity were predicted by admetSAR.

## 5. Conclusions

The bioactives and mechanism of *M. alba* L. leaves were firstly investigated through network pharmacology. The finding of this research suggested that γ-Tocopherol (−7.3 kcal/mol) on AKT1 (a hub target), 4-Dehydroxy-N-(4,5-methylenedioxy-2-nitrobenzylidene)tyramine (−8.4 kcal/mol) on PRKCA, and Lanosterol acetate (−8.4 kcal/mol) on PLA2G2A had the highest MDT, on each target. The five compounds associated with PLA2G4A did not manifest a valid MDT score. Thus, bioactives and target proteins of *M. alba* L. leaves against gout were connected to three target proteins. Hence, the three compounds, particularly γ-Tocopherol and AKT1, were regarded as the most significant bioactive and a hub target, respectively. Moreover, the promising mechanism of *M. alba* L. leaves against gout were connected to 17 signaling pathways, and a hub mechanism against gout might be to inhibit anti-arthritis immunity in synoviocytes by blocking the RAS signaling pathway. Overall, this research provides scientific evidence to support the therapeutic efficacy of *M. alba* L. leaves on gout and expounds new insights of bioactives, interactive target proteins, and mechanism(s) of *M. alba* L. leaves against gout.

## Figures and Tables

**Table 1:** A list of the identified 36 chemical compounds from *M. alba* L. leaves through GC-MS.

No.	Compound Name	Retention Time (min)	Area (%)	Pubchem ID	Pharmacological Activities (Reference)
1	Propanal, 2,3-dihydroxy-	3.702	0.16	751	No activities [27]
2	4-Oxopentyl formate	4.298	0.45	536673	No activities [27]
3	Piperazine, 2,5-dimethyl-, cis-	4.52	0.34	7816	No activities [27]
4	2,3-Dihydro-3,5-dihydroxy-6-methyl-4h-pyran-4-one	4.779	3.14	119838	Antioxidant [28]
5	2-Vinyl-9-[.beta.-d-ribofuranosyl]hypoxanthine	5.164	0.25	135493011	No activities [27]
6	Thiocyanic acid, 2-propynyl ester	5.471	3.94	123411	Anticytotoxicity [29]
7	2-Acetamidoacrylic acid	6.471	0.47	79482	No activities [27]
8	L-Cytidine	7.125	16.32	122948	No activities [27]
9	6-Amino-1-.beta.-d-ribofuranosylimidazo[4 ,5-c]pyridin-4(5H)-one	7.596	0.7	545638	No activities [27]
10	Kinic acid	7.904	12.09	1064	Cox-2 inhibitor [27]
11	2-t-Butyl-4-methyl-5-oxo-[1,3]dioxolane-4-carboxylic acid	8.077	14.34	545703	No activities [27]
12	1-(4-Bromobutyl)-2-piperidinone	8.404, 8.558, 8.750	5.15	536377	No activities [27]
13	Palmitic acid	8.914, 9.164	4.71	985	Antibacterial [27]
14	Phytol	9.471	2.49	145386	Antitumor [30]
15	Linoleoyl chloride	9.606	4.76	9817754	Anti-arteriosclerosis [31]
16	Cholestane, 4,5-epoxy-, (4.α.,5.α.)-	10.231	2.39	537014	No activities [27]
17	Tricosanoic acid	10.5	0.7	17085	No activities [27]
18	1,2,3,4-Tetrahydro-9-methyl-6-cyclohexyl-1-carbazolone	10.712	0.36	535444	No activities [27]
19	1-Palmitoylglycerol	10.923	3.57	14900	No activities [27]
20	2-Linoleoylglycerol (beta-Monolinolein)	11.702	1.14	5365676	Anti-breast cancer [32]
21	cis,cis,cis-7,10,13-Hexadecatrienal	11.741	1.27	5367366	Antibacterial [33]
22	Cholesteryl propionate	11.808	1.44	313255	No activities [27]
23	Curan-17-oic acid, 2,16-didehydro-20-hydroxy-19-oxo-, methyl ester	12.241	0.43	550468	Anti-yeast [34]
24	4-[6-[2-(4-aminophenyl)-3H-benzimidazol-5-yl]-1H-benzimidazol-2-yl]aniline	13.818	0.12	1365597	No activities [27]
25	γ-Tocopherol	13.962	0.85	14986	Antioxidant [35]
26	α-Tocopherol	14.664	1.95	14985	Antioxidant [35]
27	4-Hydroxywarfarin	15.991	0.52	54682146	No activities [27]
28	Stigmasta-5,22-dien-3-ol	16.279	0.82	6432745	Antiviral [36]
29	Clionasterol	17.096	5.48	457801	Anticomplementary [37]
30	Epicholestrol	17.779	0.36	304	No activities [27]
31	4-Dehydroxy-N-(4,5-methylenedioxy-2-nitrobenzylidene)tyramine	17.846	0.25	610062	Antibacterial [38]
32	Lupeol	18.529	1.58	259846	Anticancer, Antiviral [27]
33	Lanosterol acetate	19	3.4	3036237	No activities [27]
34	Dihydroagarofuran	19.212	0.43	21593552	Neuroprotective [39]
35	2-Methyl-7-phenylindole	19.721	0.3	610181	Antibacterial [40]
36	Lupenyl acetate	19.894	1.11	6432150	Skin cell proliferation [41]

PCIDB: PhytoChemical Interactions DB.

**Table 2:** Physicochemical properties of bioactives for good oral bioavailability and cell membrane permeability.

No.	Compounds	Lipinski Rules				Lipinski’s Violations	Biavailability Score	TPSA(Å^2^)
		MW	HBA	HBD	MLog P			
		<500	<10	≤5	≤4.15	≤1	>0.1	<140
1	Propanal, 2,3-dihydroxy-	90.08	3	2	−1.66	0	0.55	57.53
2	4-Oxopentyl formate	130.14	3	0	0.28	0	0.55	43.37
3	Piperazine, 2,5-dimethyl-, cis-	114.19	2	2	0.21	0	0.55	24.06
4	2,3-Dihydro-3,5-dihydroxy-6-methyl-4h-pyran-4-one	144.13	4	2	−1.77	0	0.85	66.76
5	2-Vinyl-9-[.beta.-d-ribofuranosyl]hypoxanthine	294.26	7	4	−1.77	0	0.55	133.49
6	Thiocyanic acid, 2-propynyl ester	97.14	1	0	1.98	0	0.55	44.45
7	2-Acetamidoacrylic acid	129.11	3	2	−0.63	0	0.85	66.40
8	L-Cytidine	243.22	6	4	−2.29	0	0.55	130.83
9	6-Amino-1-.beta.-d-ribofuranosylimidazo[4,5-c]pyridin-4(5H)-one	282.25	6	5	−2.51	0	0.55	146.62
10	Kinic acid	192.17	6	5	−2.14	0	0.56	118.22
11	2-t-Butyl-4-methyl-5-oxo-[1,3]dioxolane-4-carboxylic acid	2012.2	5	1	0.43	0	0.85	72.83
12	1-(4-Bromobutyl)-2-piperidinone	234.13	1	0	1.93	0	0.55	20.31
13	Palmitic acid	256.42	2	1	4.19	1	0.85	37.30
14	Phytol	296.53	1	1	5.25	1	0.55	20.23
15	Linoleoyl chloride	298.89	1	0	4.82	1	0.55	17.07
16	Cholestane, 4,5-epoxy-, (4.α.,5.α.)-	386.65	1	0	6.48	1	0.55	12.53
17	Tricosanoic acid	354.61	2	1	5.79	1	0.85	37.30
18	1,2,3,4-Tetrahydro-9-methyl-6-cyclohexyl-1-carbazolone	281.39	1	1	3.51	0	0.55	22.00
19	1-Palmitoylglycerol	330.5	4	2	3.18	0	0.55	66.76
20	2-Linoleoylglycerol (beta-Monolinolein)	354.52	4	2	3.42	0	0.55	66.76
21	cis,cis,cis-7,10,13-Hexadecatrienal	234.38	1	0	4.01	0	0.55	17.07
22	Cholesteryl propionate	442.72	2	0	6.7	1	0.55	26.30
23	Curan-17-oic acid, 2,16-didehydro-20-hydroxy-19-oxo-, methyl ester	354.4	5	2	1.17	0	0.55	78.87
24	4-[6-[2-(4-aminophenyl)-3H-benzimidazol-5-yl]-1H-benzimidazol-2-yl]aniline	416.48	2	4	3.34	0	0.55	109.40
25	γ-Tocopherol	416.68	2	1	5.94	1	0.55	29.46
26	α-Tocopherol	430.71	2	1	6.14	1	0.55	29.46
27	4-Hydroxywarfarin	324.33	5	2	1.95	0	0.55	87.74
28	Stigmasta-5,22-dien-3-ol	412.69	1	1	6.62	1	0.55	20.23
29	Clionasterol	414.71	1	1	6.73	1	0.55	20.23
30	Epicholestrol	386.65	1	1	6.34	1	0.55	20.23
31	4-Dehydroxy-N-(4,5-methylenedioxy-2-nitrobenzylidene)tyramine	298.29	5	0	1.49	0	0.55	76.64
32	Lupeol	426.72	1	1	6.92	1	0.55	20.23
33	Lanosterol acetate	468.75	2	0	6.98	1	0.55	26.30
34	Dihydroagarofuran	222.37	1	0	3.81	0	0.55	9.23
35	2-Methyl-7-phenylindole	207.27	0	1	3.32	0	0.55	15.79
36	Lupenyl acetate	468.75	2	0	7.08	1	0.55	26.30

MW, Molecular Weight (g/mol); HBA, Hydrogen Bond Acceptor; HBD, Hydrogen Bond Donor; LogP, Lipophilicity; Bioavailability Score, the ability of a drug or other substance to be absorbed and used by the body; TPSA (Topological Polar Surface Area).

**Table 3:** The degree value of 60 target proteins.

No.	Gene Symbol	Degree	No.	Gene Symbol	Degree
1	AKT1	31	31	CYP17A1	6
2	GAPDH	30	32	ADK	5
3	ESR1	18	33	PLA2G4A	5
4	CCND1	14	34	PLG	5
5	TLR4	14	35	PNP	5
6	AR	13	36	SHH	5
7	CYP19A1	12	37	SCD	5
8	ABCB1	10	38	PRKCA	4
9	CNR1	10	39	SPHK2	4
10	PPARG	10	40	ACP1	3
11	ADA	9	41	CA2	3
12	HMGCR	9	42	DHODH	3
13	HSPA5	9	43	EHMT2	3
14	ADORA3	8	44	GPBAR1	3
15	HSPA8	8	45	MIF	3
16	PARP1	8	46	RARB	3
17	VDR	8	47	TYMP	3
18	CHEK	7	48	EHMT1	2
19	DNMT3B	7	49	NOD1	2
20	TRPV1	7	50	PRF1	2
21	ESR2	7	51	PTGER2	2
22	GABBR1	7	52	PTPN2	2
23	PPARA	7	53	PARP2	2
24	PTGER4	7	54	HSD11B2	2
25	S1PR1	7	55	RORC	2
26	S1PR3	7	56	EBP	1
27	SHBG	7	57	PLA2G2A	1
28	ADORA2A	6	58	RORA	1
29	CDA	6	59	SLC22A6	1
30	CNR2	6	60	PPARD	1

**Table 4:** Target proteins in 17 signaling pathways enrichment related to gout.

KEGG ID & Description	Target Proteins	False Discovery Rate
hsa04917:Prolactin signaling pathway	AKT1,CCND1,ESR1,ESR2,CYP17A1	0.0004
hsa04370:VEGF signaling pathway	AKT1,SPHK2,PRKCA,PLA2G4A	0.0019
hsa04152:AMPK signaling pathway	AKT1,CCND1,PPARG,SCD,HMGCR	0.0019
hsa04071:Sphingolipid signaling pathway	AKT1,S1PR1,S1PR3,SPHK2,PRKCA	0.0019
hsa04915:Estrogen signaling pathway	AKT1,GABBR1,ESR1,ESR2,HSPA8	0.0022
hsa03320:PPAR signaling pathway	PPARA,PPARG,PPAR,SCD	0.0024
hsa04066:HIF-1 signaling pathway	AKT1,GAPDH,TLR4,PRKCA	0.0045
hsa04919:Thyroid hormone signaling pathway	AKT1,CCND1,ESR1	0.0069
hsa04664:Fc epsilon RI signaling pathway	AKT1,PRKCA,PLA2G4A	0.012
hsa04072:Phospholipase D signaling pathway	AKT1,SPHK2,PRKCA,PLA2G4A	0.012
hsa04933:AGE-RAGE signaling pathway in diabetic complications	AKT1,CCND1,PRKCA	0.0232
hsa04024:cAMP signaling pathway	AKT1,PPARA,GABBR1	0.0232
hsa04014:Ras signaling pathway	AKT1,PRKCA,PLA2G2A,PLA2G4A	0.0318
hsa04068:FoxO signaling pathway	AKT1,CCND1,S1PR1	0.0391
hsa04371:Apelin signaling pathway	AKT1,CCND1,SPHK2	0.0408
hsa04340:Hedgehog signaling pathway	SHH,CCND1	0.0416
hsa04310:Wnt signaling pathway	CCND1,PRKCA,PPARA	0.0468

**Table 5:** Binding energy and interactions of potential bioactives on AKT1 (PDB ID: 4GV1).

						Hydrogen Bond Interactions		Hydrophobic Interactions
Protein	Ligand	PubChem ID	Symbol	Binding Energy (kcal/mol)	Amino Acid Residue	R Group(s) Involved in Hydrogen Boding	Distance (Å)	Amino Acid Residue
4GV1	γ-Tocopherol	14986	A1	−7.3	N/A	N/A	N/A	Thr312,Asp274, Asp292
								Leu295, Gly294, Phe161
								His194, Glu191
	α-Tocopherol	14985	A2	−7.0	Thr160	R-OH	2.80, 3.08	Gly159, Lys276, Asp292
								His194, Leu295, Glu191
								Asp274, Thr312, Gly311
								Asn279, Phe161
	1- Palmitoylglycerol	14900	A3	−6.9	Asp274	R-OH	2.97	Leu295, Thr160, Gly159
					Asp292		3.03	Phe161
					Gly294		2.95	
	cis-cis-cis-7,10,13 Hexadecatrienal	5367366	A4	−4.8	Ser240	Aldehyde	2.89	Phe236, Tyr350, Leu347
								Arg346, Gly345, Glu341
								Leu239

**Table 6:** Binding energy and interactions of potential bioactives on PRKCA (PDB ID: 3IW4).

						Hydrogen Bond Interactions		Hydrophobic Interactions
Protein	Ligand	PubChem ID	Symbol	Binding Energy (kcal/mol)	Amino Acid Residue	R Group(s) Involved in Hydrogen Boding	Distance (Å)	Amino Acid Residue
3IW4	1-Palmitoylglycerol	14900	B1	−6.6	ASP-395	R-OH	2.88	Val-664,Ile667,Pro666
					Leu-393	R-OH	3.02	Pro398.Gln402
					Lys-396	R-OH, Aldehyde	2.87,3.26	
					Asn-660	R-OH	3.06	
	2-Linoleoylglycerol	5365676	B2	−6.9	Leu393	R-OH	3.04	Val-664, Pro666,Ile667
					Asp395	R-OH	3.14	Gln402,Pro398
					Lys396	R-OH, Carboalkoxy	3.25,3.26	
					Gln662	R-OH	3.06	
					Asn660	R-OH, Carbonyl	2.81,3.24	
	Linoleoyl chloride	9817754	B3	−4.8	Lys396	Haloform	3.07	Gln402, Pro398,Gln662
								Val664
	Palmitic acid	985	B4	−5.0	Lys396	Carbonyl, R-OH	2.99, 3.11	Val664, Gln662, His553
					Asp395	R-OH	3.10	Ser549, Glu552, Gln402
					Leu393	R-OH	3.15	Pro398
	Tricosanoic acid	17085	B5	−6.5	Lys396	Carbonyl, R-OH	3.20, 3.33	Gln402, Val664, Pro666
					Leu393	R-OH	2.89	Pro398
	Phytol	145386	B6	−5.6	Asp395	R-OH	3.11	Pro398, Ser549, His553
					Leu393	R-OH	3.00	Glu552, Val664, Gln402
					Lys396	R-OH	3.00	
	4-Dehydroxy-N-(4, 5-methylenedioxy-2-nitrobenzylidene) tyramine	610062	B7	−8.4	Lys-396	Nitro, Imine	3.03, 3.23	Pro398, Ile667, Val664
					Asn-660	Nitro	2.82	Glu552, Gln402, Gln662

**Table 7:** Binding energy and interactions of potential bioactives on PLA2G2A (PDB ID: 1KVO).

						Hydrogen Bond Interactions		Hydrophobic Interactions
Protein	Ligand	PubChem ID	Symbol	Binding Energy (kcal/mol)	Amino Acid Residue	R Group(s) Involved in Hydrogen Boding	Distance (Å)	Amino Acid Residue
1KVO	1-Palmitoylglycerol	14900	C1	−6.8	Tyr112	R-OH	2.06	Val3, His6, Tyr111
					Gly25	R-OH	2.32	Ser113, Cys28, Gly22
					Phe23	Ether	2.33	
					Val30	Ester	2.98	
					Asn114	R-OH	2.40, 3.23	
	Linoleoyl chloride	9817754	C2	−4.8	N/A		N/A	Tyr111, Phe23,His6
								Leu2, Phe63, Val3
	Palmitic acid	985	C3	−5.4	Cys59	R-OH	3.18	Gly60, Phe-63, Lys62
					Thr61	R-OH	2.96	Glu55, Asn1, Phe63
	Tricosanoic acid	17085	C4	−5.9	Asn114	R-OH	3.04	Leu19, Glu16, Tyr111
					Cys28	R-OH	2.99	
					Phe23	R-OH	3.15	
					Gly25	R-OH	2.29	
					Tyr112	R-OH	2.06	
	Lanosterol acetate	3036237	C5	−8.4	N/A	N/A	N/A	Asn-114, Ser-113, Phe23
								Tyr-111, Leu2, Ala18
								Val3

**Table 8:** Binding energy of potential bioactives on PLA2G4A (PDB ID: 1BCI).

						Hydrogen Bond Interactions		Hydrophobic Interactions
Protein	Ligand	PubChem ID	Symbol	Binding Energy (kcal/mol)	Amino Acid Residue	R Group(s) Involved in Hydrogen Boding	Distance (Å)	Amino Acid Residue
1BCI	2-Linoleoylglycerol	5365676	D1	−4.9	Gln83	R-OH	3.22	Tyr16, Pro54, Thr53
					Thr52	R-OH	2.90	Leu79
					Asp80	R-OH	2.87, 3.19	
	Linoleoyl chloride	9817754	D2	−4.0	Lys58	Haloform	3.04	Pro54, Ile78, Phe77
								Tyr16, Thr53
	Palmitic acid	985	D3	−3.3	His-62	R-OH	3.14	Ala94, Tyr45, Phe63
	Tricosanoic acid	17085	D4	−3.6	N/A	N/A	N/A	Tyr16, Ile78, Pro54
								Phe77
	cis-cis-cis-7,10,13 Hexadecatrienal	5367366	D5	−4.1	N/A	N/A	N/A	Asn95, Tyr96, Met98
								Glu100, Phe35, Val97
								Gly36

**Table 9:** Toxicological properties of the key bioactives on AKT1 (PDB ID: 4GV1) in the molecular docking study.

Parameters	Compound Name		
	γ-Tocopherol	4-Dehydroxy-N-(4, 5-methylenedioxy-2-nitrobenzylidene) tyramine	Lanosterol Acetate
Ames toxicity	NAT	AT	NAT
Carcinogens	NC	NC	NC
Acute oral toxicity	Ⅲ	Ⅲ	Ⅲ
Rat acute toxicity	2.1598	2.6672	2.0477

AT: Ames toxic; NAT: Non Ames toxic; NC: Non-carcinogenic; Category-II means (50 mg/kg > LD50 < 500 mg/kg); Category-III means (500 mg/kg > LD50 < 5000 mg/kg).

## Data Availability

All data generated or analyzed during this study are included in this published article (and its Supplementary Information files).

## Supplementary Materials

The following are available online at https://www.mdpi.com/article/10.3390/ijms22179372/s1.

## Abbreviations

AGE-RAGE:	Advanced Glycation End Product- Receptor of Advanced Glycation End Product;
AKT1:	AKT Serine/Threonine Kinase 1;
AMPK:	AMP-activated protein kinase;
APLN:	Apelin;
APJ:	APLN receptor;
AS:	Ankylosing Spondylitis;
cAMP:	cyclic Adenosine MonoPhosphate;
COX-2:	Cyclooxygenase-2;
FcεRI:	Fc epsilon RI;
FLS:	Fibroblast-Like Synoviocytes;
FoxO:	Forkhead box O;
GABAA:	γ-Aminobutyric acid type A;
GC-MS:	Gas Chromatography—Mass Spectrum;
GSEA:	Gene Set Enrichment Analysis;
HIF-1:	*Hypoxia Inducible Factor -1*;
HIF-1α:	Hypoxia Inducible Factor -1 Alpha;
IL-1β:	Interleukin 1 beta;
IL-17:	Interleukin-17;
IL-RA:	Interleukin Receptor Antagonist;
MDT:	Molecular Docking Test;
*M. alba*:	*Morus alba*
MSU:	Mono Sodium Urate;
NKA:	Na^+^-K^+^-ATPase;
NLRP3:	Nod-like receptor protein 3;
NSAIDs:	Non-Steroidal Anti-Inflammatory Drugs;
P2Y_14_R:	P2Y_14_ receptor;
PPAR:	Peroxisome Proliferator-Activated Receptor;
PPAR–γ:	Peroxisome Proliferator-Activated Receptor –Gamma;
PPI:	Protein-Protein Interaction
RAS:	Renin Angiotensin System;
SMILES:	Simplified Molecular Input Line Entry System;
S-T-B:	Signaling pathway- Target protein- Bioactive;
STP:	SwissTargetPrediction;
UA:	Uric Acid;
VEGF:	*Vascular Endothelial Growth Factor*;
Wnt:	*Wingless-INT*;
XO:	Xanthine Oxidase
